# Whole-cell surface-display system in attenuated *Salmonella* Enteritidis for slide microagglutination-based antibody detection

**DOI:** 10.1186/s12934-026-03083-x

**Published:** 2026-08-08

**Authors:** Yacory Sernaque-Aguilar, Dora Rios-Matos, Astrid Poma-Acevedo, Alen Zimic-Sheen, María Belén-Balta, Ricardo Choque-Guevara, Ricardo Montesinos-Millán, Mario I. Salguedo-Bohorquez, Gisela Isasi-Rivas, Angela Montalván-Avalos, Freddy Ygnacio-Aguirre, Julio Ticona, Manolo Fernández-Sánchez, Manolo Fernández-Díaz, Mirko Zimic

**Affiliations:** 1 Laboratorios de Investigación y Desarrollo, Farmacológicos Veterinarios S.A.C, Chincha, Perú; 2 Laboratorio de Bioinformática, Biología Molecular y Desarrollos Tecnológicos, Facultad de Ciencias e Ingeniería, Universidad Peruana Cayetano Heredia, Lima, Perú

**Keywords:** *Salmonella* Enteritidis, Surface display, INP, Microagglutination, Antibodies

## Abstract

**Background:**

Microagglutination is a sensitive, rapid, and cost-effective serological assay widely used for disease diagnosis and surveillance in animals and humans. However, it traditionally relies on culturing live pathogenic bacteria as antigens, posing biosafety risks and limiting its application primarily to bacterial pathogens. Bacterial surface display technology offers a promising alternative by enabling the presentation of heterologous proteins or peptides on the bacterial surface, avoiding the need to culture the target pathogen, simplifying antigen preparation, and enabling potential diagnostic applications for non-bacterial pathogens. Despite these advantages, integration of this technology with slide-based microagglutination assays has not yet been evaluated. In this proof-of-concept, we evaluated a surface display platform in attenuated *Salmonella* Enteritidis for antibody detection by slide microagglutination, using the nucleocapsid (N) protein of Infectious Bronchitis Virus (IBV) as a model antigen.

**Results:**

The IBV N protein was fused to the N-terminal anchor domain of the ice nucleation protein (INP) and cloned into pET-28a. The resulting INP_N fusion protein was expressed in attenuated *Salmonella* Enteritidis, and its surface exposure was evaluated by immunofluorescence and flow cytometry on non-permeabilized cells, with flow cytometry indicating a surface display efficiency of 96.5%. Slide microagglutination was performed using 50 hen sera and inactivated *Salmonella* Enteritidis cells presenting surface-exposed N protein. This assay identified all positive sera and 20 of 25 negative sera, showing substantial agreement with the reference ELISA (κ = 0.8), with preliminary estimates of 100% sensitivity (95% CI, 87–100%) and 80% specificity (95% CI, 61–91%). No cross-reactivity was observed with commercial antisera against pathogens unrelated to IBV.

**Conclusions:**

The attenuated *Salmonella* surface-display platform integrated into a slide microagglutination assay showed preliminary feasibility for antibody detection under the conditions evaluated. This approach does not require live pathogen cultivation, specialized equipment, antigen purification, or chemical conjugation steps. However, further optimization is needed to improve the platform’s display efficiency and enhance specificity without compromising sensitivity. In addition, evaluation using a larger and more diverse sample set, including field samples, is required to further assess the performance and potential applicability of the assay.

**Supplementary Information:**

The online version contains supplementary material available at https://doi.org/10.1186/s12934-026-03083-x.

## Background

Microagglutination is a sensitive, rapid, cost-effective, and simple serological assay [1, 2] used for the presumptive detection of infectious agents in animals and humans [3]. The assay relies on the formation of cellular aggregates resulting from the interaction between antibodies in convalescent serum and bacterial surface antigens [4, 5]. Although methods such as the enzyme-linked immunosorbent assay (ELISA) provide greater sensitivity and specificity, microagglutination remains used for surveillance and screening, particularly in resource-limited laboratories [6, 7].

However, conventional microagglutination assay is restricted to the detection of bacterial infections and relies on live pathogen cultures, which pose biosafety risks. To overcome these limitations, alternative assays using latex microspheres or other antigen-coated solid supports have been developed, enabling antibody detection against both bacterial and viral pathogens while improving visualization and reducing biosafety risks [8, 9]. Nevertheless, these approaches require purified antigens or antibodies and their chemical conjugation to solid supports, increasing assay complexity and cost [10] and limiting their accessibility.

Bacterial surface display systems offer an attractive alternative by exposing heterologous peptides or proteins on bacterial surfaces. This strategy eliminates the need for protein purification and complex conjugation steps, thereby simplifying assay development and reducing production costs [11–13]. In these systems, proteins or peptides are fused to anchor motifs, typically cell surface proteins or their fragments, facilitating their engineering and application [14, 15].

A wide range of anchor motifs can be found in Gram-negative bacteria [16]. Among them, the INP-based anchor is a strong candidate for surface display systems due to its stable expression, efficient membrane translocation, and modular central repeat domain (CRD), which allows control over fusion protein length [14]. Furthermore, previous studies have reported that INP expression does not significantly affect membrane integrity or host growth [17], and has been used to display heterologous proteins in several bacterial species, including *Escherichia coli* [18], *Salmonella* Typhi [19], *Vibrio anguillarum* [20], and *Pseudomonas syringae* [21].

Attenuated *Salmonella* strains are considered safe, easy to handle and inexpensive vectors for the expression of not only cytoplasmic but also secreted or surface exposed recombinant proteins [22]. In particular, the attenuated *S.* Enteritidis ΔXIII strain, which carries deletions in the genes encoding 12 GGDEF family proteins and the rpoS gene, has been shown to be avirulent and to exhibit reduced environmental persistence, allowing its safe use as a biological platform [23, 24].

Bacterial surface display systems have been widely applied in biosorbent development [25], vaccines, peptide library screening, whole-cell biocatalysts, and biosensors for diagnostic, industrial, and environmental applications [26]. In serological diagnostics, these systems have been successfully incorporated into enzyme-linked immunosorbent assay (Display-ELISA) for antibody detection against systemic lupus erythematosus [27], bovine αS1-casein [28], and hepatitis C virus [29], as well as into lateral flow immunoassays (LFIAs) for β-galactosidase detection [30]. However, their application has been largely restricted to immunoassay formats that rely on immobilization on solid supports or require specialized detection systems. To date, bacterial surface display systems have not been explored as antigen display platforms for slide microagglutination assays. The integration of surface display technology with the inherent simplicity of slide microagglutination-based serology could eliminate the need for antigen purification and immobilization steps, thereby streamlining assay preparation.

In this study, we developed a whole-cell surface display platform based on attenuated *S.* Enteritidis and applied it in a slide microagglutination assay for antibody detection. As a proof of concept, the infectious bronchitis virus (IBV) nucleocapsid (N) protein was selected as the model antigen because of its suitability for early serological diagnosis. This suitability is attributed to its high expression during infection, strong immunogenicity, and high degree of conservation among IBV strains. IBV is a pathogen of major economic importance to the global poultry industry, causing respiratory and nephropathogenic disease in broilers and reducing egg production in laying hens and breeder flocks [31–35]. The assay was evaluated against a commercial anti-IBV ELISA as the reference method. This study highlights the potential of applying bacterial surface display technology to microagglutination-based immunoassays.

## Materials and methods

### Bacterial strains and culture conditions

The attenuated *S.* Enteritidis strain 3934vac (Δgenes encoding GGDEF-domain proteins, Δ*rpoS*, and Δ*waaL*) was used for the expression of the fusion protein (INP_N). *E. coli* DH5α (Invitrogen, USA) was used in plasmid maintenance and amplification. Both recombinant strains were cultured in Luria-Bertani (LB) medium supplemented with kanamycin (50 µg/mL) and incubated at 37 °C with shaking at 200 rpm.

### Expression cassette design and recombinant strain screening

The INP_N expression cassette was designed using the amino acid sequences of the INP from *P. syringae* (UniProt: O30611) and the N protein from the IBV Beaudette strain (UniProt: P69596). The cassette included only the INP N-terminal region (first 191 amino acids), which serves as a membrane anchor, while the CRD and C-terminal region were replaced by the full-length IBV N protein. A flexible linker (GGGSGGGS) was included between the INP anchor and the N protein. In addition, a six-histidine tag (6xHis tag) was added in the C-terminal region of the fusion protein. The coding sequence of the fusion protein was optimized for expression in *Salmonella* using the Java Codon Adaptation Tool (JCAT). INP_N expression was placed under the control of the PR promoter from bacteriophage lambda, excluding the sequence encoding the cI857 repressor, to allow constitutive expression of the fusion protein [36]. The expression cassette was synthesized and cloned into the pET-28a (+) vector by GenScript, yielding the plasmid pET_INP_N.

For recombinant strain verification, plasmid DNA was extracted from cultures of *Salmonella* strains harboring pET-28a (*S.* pET-28a) and pET_INP_N (*S.* pET_INP_N) using the PureLink™ HiPure Plasmid Miniprep Kit (Invitrogen, USA), according to the manufacturer’s protocol. The expression cassette was then amplified from the purified plasmid using the Q5 High-fidelity 2X Master Mix (New England Biolabs, USA) and primers flanking the cassette: 5’ CATACCCACGCCGAAACAA 3’ and 5’ GCTTAATGCGCCGCTACAG 3’. PCR conditions were: initial denaturation at 98 °C for 30 s, followed by 35 cycles of 98 °C for 10 s, 66 °C for 10 s, 72 °C for 25 s, with a final extension at 72 °C for 2 min. Amplified products were analyzed by electrophoresis on a 1% agarose gel and visualized using a CCD camera on an Azure c600 imaging system (Azure Biosystems, USA).

### In silico modelling and structural alignment

The three-dimensional structure of the extracellular region of the INP_N fusion protein was predicted using AlphaFold 3 with default server parameters. The input sequence comprised 544 amino acids (aa), including the last 121 aa of the INP N-terminal domain, a flexible linker, the full-length IBV N protein, and a C-terminal 6×His tag. AlphaFold 3 generated five structural models, and the top-ranked model based on the predicted template modeling (pTM) and interface predicted template modeling (ipTM) scores was selected for subsequent analysis. Structural quality was assessed using the QMEANDisCo global score, the ProSA-web Z-score, and Ramachandran plot analysis generated by PDBsum. Model reliability was considered acceptable when QMEANDisCo scores were close to 1, ProSA-web Z-scores fell within the range of experimentally determined structures of similar size, and more than 90% of residues were located in favored regions of the Ramachandran plot, according to the validation criteria recommended by Swiss-Model and ProSA-web [37–39].

The region corresponding to the IBV N protein in the predicted model was structurally aligned with the experimentally determined N-terminal (PDB ID: 2GEC) and C-terminal (PDB ID: 2GE7) domains of the IBV N protein obtained from the Protein Data Bank (PDB). Structural alignment was performed using UCSF Chimera v1.17.3, and alignment quality was evaluated with TM-align based on root mean square deviation (RMSD) and TM-score values.

### Preparation of electrocompetent cells and electroporation

Electrocompetent cells were prepared following the protocol established by Miller and Nickoloff [40], with slight modifications. Briefly, *Salmonella* 3934vac was thawed, inoculated into LB medium and incubated at 37 °C for 15 h at 200 rpm. Subsequently, 500 µL of the reactivated culture were diluted into 50 mL of LB medium and incubated under the same conditions to reach an optical density at 600 nm (OD_600_) of ~ 0.567. The culture was then placed on ice for 20 min and centrifuged at 4,000 × g for 15 min at 4 °C. The cell pellet was sequentially resuspended in 50 mL, 25 mL, and 2 mL of cold 10% glycerol, with centrifugation at 4,000 × g for 15 min at 4 °C performed after each resuspension. Finally, the cells were resuspended in 200 µL of cold 10% glycerol and aliquoted in 100 µL volumes into 1.5 mL microtubes.

For electroporation, 200 ng of pET-28a and pET_INP_N plasmids were added independently in tubes containing 100 µL of electrocompetent cells and the mixture was transferred to a 0.2 cm cold electroporation cuvette. Electroporation was performed using a Gene Pulser XCell (Bio-Rad, USA) at 2 500 V for 5 ms. Immediately 900 µL of SOC medium (Invitrogen, USA) was added, the cells were transferred to a sterile 1.5 mL microtube and incubated at 37 °C for 1 h at 200 rpm. Cells were then centrifuged at 3,500 × g for 3 min at room temperature (RT) and the pellet was resuspended in 150 µL of LB. Finally, 100 µL of culture was seeded on LB agar supplemented with kanamycin (50 µg/mL) and the plates were incubated at 37 °C for 18–20 h.

### Growth analysis of wild type and recombinant strains

Wild type (WT) and recombinant *Salmonella* strains were reactivated on LB agar plates supplemented with kanamycin (50 µg/mL) and incubated at 37 °C for ~ 20 h. One colony of each strain was inoculated into 4 mL of LB broth with kanamycin (50 µg/mL) and incubated at 37 °C for 16 h at 200 rpm. Then, 50 µL of the overnight culture was placed in 50 mL of LB broth with kanamycin (50 µg/mL) and an aliquot of 400 µL was extracted for evaluation as initial time (T0). The culture was incubated at 37 °C under agitation at 200 rpm and 400 µL aliquots were collected hourly over a 10 h period (T1-T10). From the extracted aliquot, 8 serial dilutions were performed at 1:10 factor and three replicates of each dilution were seeded on LB agar with kanamycin (50 µg/mL) applying the drop plate method (10 µL in each drop). Plates were allowed to dry for 10 min at RT and incubated at 37 °C for 24 h. Colonies were subsequently counted, and the number of colony-forming units per milliliter (CFU/mL) were calculated.

### Total protein extraction

For total protein extraction, 5 mL of *S.* pET-28a and *S.* pET_INP_N cultures were centrifuged at 11,000 rpm for 10 min at 4 °C and the pellet was resuspended in urea lysis buffer containing 8 M urea, 10% glycerol, 20% SDS, 1 M Dithiothreitol (DTT), 1.5 M Tris pH 6.8 and 1 mM phenylmethylsulfonyl fluoride (PMSF). The samples were incubated with agitation at RT for 90 min. Then, 0.5 µL of Pierce Universal Nuclease Cell Lysis (Thermo Fisher Scientific, USA) was added and incubated in agitation at RT for 45 min. Total protein extracts were stored at -20 °C.

### Western blot analysis

For detection of the fusion protein by immunoblotting, total lysates were diluted 1:10 and 20 µL of each sample was loaded per well onto two 4–20% gradient ExpressPlus™ PAGE gels (GenScript, USA). Electrophoresis was performed at 60 V for 40 min, followed by 110 V for 1.5 h. Proteins were then transferred onto two eBlot L1 nitrocellulose membranes using the eBlot™ L1 system (GenScript, USA). Membranes were washed with 1× TBST (0.1% Tween-20 in 1× TBS, pH 7.4) for 10 min and blocked overnight at 4 °C with 5% non-fat milk in PBS containing 0.1% Tween-20 (PBS-T). After blocking, membranes were washed four times with TBST (5 min each). One membrane was incubated with HRP-conjugated anti-His antibody (GenScript, USA; 1:2500 dilution), while the second membrane was incubated with commercial chicken anti-IBV serum (Charles River, USA; 1:250 dilution), both prepared in 5% non-fat milk and incubated for 4 h at RT. Membranes were subsequently washed four times with TBST (10 min each). The membrane probed with chicken anti-IBV serum was further incubated for 2 h with HRP-conjugated anti-chicken IgY secondary antibody (GenScript, USA; 1:5000 dilution) prepared in 5% non-fat milk. After a final series of four TBST washes, both membranes were incubated with Radiance Plus chemiluminescent substrate (Azure Biosystems, USA) for 2 min. Chemiluminescent signals were detected using an Azure C600 imaging system (Azure Biosystems, USA). Precision Plus Protein™ WesternC molecular weight marker (Bio-Rad, USA) was used according to the manufacturer’s instructions.

### Flow cytometry analysis

Cultures of *S.* pET-28a and *S.* pET_INP_N were grown to an OD_600_ of 0.8–0.9. Then ~ 9 mL of each culture were harvested by centrifugation at 1,500 × g for 5 min at 4 °C. Cell pellets were washed twice with 9 mL of Dulbecco’s phosphate-buffered saline (DPBS), resuspended, and aliquoted into 1.5 mL micro-centrifuge tubes (1 mL per tube). After a second centrifugation step, cells were fixed in 200 µL of 2% paraformaldehyde (PFA) in PBS for 15 min at 4 °C. Fixation was followed by sequential washes with 1 mL and 500 µL of FACS buffer (DPBS supplemented with 1% BSA).

For surface labeling, cells were incubated with a primary antibody anti-flagellin (Abcam, USA; 1:200 in 50 µL FACS buffer) and anti-His AF647^®^ conjugated antibody (GenScript, USA; 1:200 in 100 µL FACS buffer) for 2 h at 37 °C with gentle agitation. After two washes with 1 mL of DPBS, cells were incubated for 1 h at RT with an Alexa Fluor 488-conjugated secondary antibody (Abcam, USA; 1:100 in DPBS). Following final washing steps, pellets were resuspended in 500–600 µL of DPBS for acquisition.

Flow cytometry data were collected using a Cytek Northern Lights™spectral flow cytometer (Cytek Biosciences, USA). Instrument settings were optimized using single-stained and fluorescence-minus-one (FMO) controls, 50,000 events per sample were acquired, and bacterial populations were gated based on forward and side scatter properties to exclude debris and aggregates.

### Indirect immunofluorescence analysis (IFA)

Cryopreserved *S.* pET_INP_N and *S.* pET-28a strains were reactivated on LB agar with kanamycin (50 µg/mL) and incubated at 37 °C. Individual colonies were inoculated into 5 mL of LB broth with kanamycin (50 µg/mL) and incubated at 37 °C, 200 rpm, until an OD_600_ of 0.87-1.0 was reached. Approximately 9 × 10^7^ cells were harvested by centrifugation at 1,500 × g for 5 min, washed with 1 mL of 1X PBS, and fixed in 200 µL of 1% PFA in PBS at 4 °C for 15 min. Cells were collected by centrifugation, washed twice with 1 mL and 500 µL of PBS, and 100 µL of the suspension was applied to poly-L-lysine coated slides for 20 min. The cells were blocked with 3% non-fat milk for 1 h at RT, washed with PBS, and incubated for 2 h with monoclonal anti-His antibody (GenScript, USA) diluted 1:500 in 1% BSA. After washing, the cells were incubated with Alexa Fluor 488 secondary antibody (Abcam, USA) for 1 h at RT. Cells were washed, and each sample was analyzed on a ZEISS Axioscope 5 (Carl Zeiss, Germany) at 1000× magnification. Digital images were captured.

### Serum samples

Fifty chicken serum samples (25 IBV-positive and 25 IBV-negative, determined by the reference commercial ELISA) were analyzed in this proof-of-concept study. Serum samples were previously collected from specific pathogen-free (SPF) hens that had been immunized intranasally with a commercial Massachusetts strain vaccine (BRONQUITIS F 125 PLUS^®^, FARVET S.A.C., Peru) according to the manufacturer’s instructions and stored at − 20 °C. The collection of these samples was approved by the Institutional Ethics Committee for the Use of Animals of the Universidad Peruana Cayetano Heredia (CIEA-E-021-05-24).

### Anti-IBV antibody detection by ELISA

The ELISA test was used as the reference method for detecting seropositivity, following the manufacturer’s instructions of the commercial IBV ELISA kit (BioChek, The Netherlands). Briefly, 100 µL each of the 50 chicken serum samples (diluted 1:500), along with positive and negative controls, were added to wells pre-coated with inactivated IBV antigen. After 30 min incubation at RT, the contents of the wells were aspirated and washed four times with 350 µL of wash buffer. Subsequently, 100 µL of anti-chicken IgG conjugate was added, and the plate incubated for another 30 min at RT. After washing, 100 µL of p-nitrophenyl phosphate substrate was added, followed by a 30 min incubation at RT. The reaction was stopped with 100 µL of sodium hydroxide stop solution, and absorbance was read within 30 min on the EPOCH-2 spectrophotometer (Biotek) at a wavelength of 450 nm.

### Antigen preparation and microagglutination assay

For antigen preparation, *S.* pET_INP_N was reactivated on LB agar containing kanamycin (50 µg/mL) and incubated at 37 °C for 24 h. Colonies were inoculated into 5 mL of LB broth with kanamycin (50 µg/mL) and incubated at 37 °C, until an OD_600_ of 0.9-1 was reached. Cultures was centrifuged at 6,000 × g for 3 min at RT, and the pellet was washed with 500 µL of 1X DPBS. Cells were inactivated with 0.1% PFA at 4 °C for 15 min, followed by centrifugation at 6,000 × g for 3 min at RT and sequentially washed with 200 µL of 1X DPBS and 200 µL of Milli-Q water. The final pellet was resuspended in 200 µL of Milli-Q water and used as antigen in the microagglutination assays.

Undiluted serum samples and their respective dilutions, were initially evaluated to determine the conditions that allowed optimal discrimination between positive and negative samples. Based on this analysis, undiluted serum was established for the microagglutination assays. For the assay, 20 µL of the previously prepared antigen were placed onto a glass slide, followed by the addition of 20 µL of serum. The final mixture (40 µL) was incubated at RT for 90 s with gentle circular agitation. Samples exhibiting strong agglutination, characterized by the formation of large bacterial aggregates, were considered positive, whereas those showing no aggregates or only small granules were considered as negative. Microagglutination patterns were evaluated by optical microscopy at 400× magnification.

All serum samples were evaluated by microagglutination in a blinded manner, without prior knowledge of the corresponding ELISA results.

### Cross reactivity analysis

To evaluate potential cross reactivity of *S.* pET_INP_N with pathogens associated with infectious bronchitis and other major avian diseases, a panel of nine commercial chicken antisera was tested using a slide microagglutination assay, with *S.* pET_INP_N used as the antigen. The antisera were specific to Fowl Adenovirus Type I (10100384, Charles River, Norwich, CT, USA), Chicken Anemia Virus (10100498, Charles River, Norwich, CT, USA), Fowlpox Virus (10100430, Charles River, Norwich, CT, USA), *Mycoplasma synoviae* (10100482, Charles River, Norwich, CT, USA), Avian Influenza A (H5N2) Virus (10101644, Charles River, Norwich, CT, USA), Infectious Laryngotracheitis Virus (10100470, Charles River, Norwich, CT, USA), Newcastle Disease Virus (10100486, Charles River, Norwich, CT, USA), Marek’s Disease Virus (10100474, Charles River, Norwich, CT, USA) and Infectious Bursal Disease Virus (10100462, Charles River, Norwich, CT, USA). In addition, a commercial chicken antiserum specific to IBV (Massachusetts strain) (10100454, Charles River, Norwich, CT, USA) was also evaluated to verify positive microagglutination patterns.

### Detection of anti-*Salmonella* antibodies

To rule out that the *S.* pET_INP_N microagglutination observed with IBV-positive sera was due to the presence of anti-*Salmonella* antibodies, a *Salmonella*-specific assay was performed. For this purpose, a commercial anti-*Salmonella* ELISA kit (BioChek, The Netherlands) was used, strictly following the manufacturer’s instructions. Briefly, 100 µL of each IBV-positive chicken serum sample (diluted 1:100), along with positive and negative controls, was added to wells pre-coated with inactivated group D lipopolysaccharide (LPS) antigen. After 30 min incubation at RT, the contents of the wells were aspirated and washed four times with 350 µL of wash buffer. Subsequently, 100 µL of anti-chicken IgG conjugate was added, and the plate was incubated for another 30 min at RT. After washing, 100 µL of p-nitrophenyl phosphate substrate was added, followed by a 15 min incubation at RT. The reaction was stopped with 100 µL of sodium hydroxide stop solution, and absorbance was read within 30 min on the EPOCH-2 spectrophotometer (Biotek) at a wavelength of 450 nm. Samples were considered positive when the S/P ratio was ≥ 0.2.

### Statistical analysis

The statistical significance of growth differences between recombinant *Salmonella* strains and the WT strain was assessed using a two-way analysis of variance (ANOVA), followed by Dunnett’s multiple comparisons post hoc test, implemented in GraphPad Prism version 6.1 (GraphPad Software, USA). A *p*-value < 0.05 was considered statistically significant.

Agreement between ELISA and microagglutination tests was assessed with Cohen’s kappa index (k). Diagnostic performance metrics, including sensitivity, specificity, positive predictive value (PPV), and negative predictive value (NPV), were calculated with their corresponding 95% confidence intervals (95% CI) from a 2 × 2 contingency table based on dichotomized results from both assays. ELISA was considered as the reference assay for evaluating the diagnostic performance of the microagglutination assay. These analyses were performed in Python using standard libraries (*statsmodels*,* pandas* and *scikit-learn*).

Sensitivity, specificity, positive and negative predicted value were calculated by applying the following formulas:

$$\:\mathrm{\%}\:\mathrm{S}\mathrm{e}\mathrm{n}\mathrm{s}\mathrm{i}\mathrm{v}\mathrm{i}\mathrm{t}\mathrm{y}=\frac{\mathrm{t}\mathrm{r}\mathrm{u}\mathrm{e}\:\mathrm{p}\mathrm{o}\mathrm{s}\mathrm{i}\mathrm{t}\mathrm{i}\mathrm{v}\mathrm{e}}{(\mathrm{t}\mathrm{r}\mathrm{u}\mathrm{e}\:\mathrm{p}\mathrm{o}\mathrm{s}\mathrm{i}\mathrm{t}\mathrm{i}\mathrm{v}\mathrm{e}+\mathrm{f}\mathrm{a}\mathrm{l}\mathrm{s}\mathrm{e}\:\mathrm{n}\mathrm{e}\mathrm{g}\mathrm{a}\mathrm{t}\mathrm{i}\mathrm{v}\mathrm{e})}\:\mathrm{X}\:100$$

$$\:\mathrm{\%}\:\mathrm{S}\mathrm{p}\mathrm{e}\mathrm{c}\mathrm{i}\mathrm{f}\mathrm{i}\mathrm{c}\mathrm{i}\mathrm{t}\mathrm{y}=\frac{\mathrm{t}\mathrm{r}\mathrm{u}\mathrm{e}\:\mathrm{n}\mathrm{e}\mathrm{g}\mathrm{a}\mathrm{t}\mathrm{i}\mathrm{v}\mathrm{e}}{(\mathrm{f}\mathrm{a}\mathrm{l}\mathrm{s}\mathrm{e}\:\mathrm{p}\mathrm{o}\mathrm{s}\mathrm{i}\mathrm{t}\mathrm{i}\mathrm{v}\mathrm{e}+\mathrm{t}\mathrm{r}\mathrm{u}\mathrm{e}\:\mathrm{n}\mathrm{e}\mathrm{g}\mathrm{a}\mathrm{t}\mathrm{i}\mathrm{v}\mathrm{e})}\:\mathrm{X}\:100$$

$$\:\mathrm{P}\mathrm{P}\mathrm{V}=\frac{\mathrm{t}\mathrm{r}\mathrm{u}\mathrm{e}\:\mathrm{p}\mathrm{o}\mathrm{s}\mathrm{i}\mathrm{t}\mathrm{i}\mathrm{v}\mathrm{e}}{\mathrm{a}\mathrm{l}\mathrm{l}\:\mathrm{p}\mathrm{o}\mathrm{s}\mathrm{i}\mathrm{t}\mathrm{i}\mathrm{v}\mathrm{e}\:\mathrm{t}\mathrm{e}\mathrm{s}\mathrm{t}}\:\mathrm{X}\:100$$

$$\:\mathrm{N}\mathrm{P}\mathrm{V}=\frac{\mathrm{t}\mathrm{r}\mathrm{u}\mathrm{e}\:\mathrm{n}\mathrm{e}\mathrm{g}\mathrm{a}\mathrm{t}\mathrm{i}\mathrm{v}\mathrm{e}}{\mathrm{a}\mathrm{l}\mathrm{l}\:\mathrm{n}\mathrm{e}\mathrm{g}\mathrm{a}\mathrm{t}\mathrm{i}\mathrm{v}\mathrm{e}\:\mathrm{t}\mathrm{e}\mathrm{s}\mathrm{t}}\:\mathrm{X}\:100$$

## Result

### Construction of the INP_N cassette and fusion protein expression in *Salmonella*

**Fig. 1:**
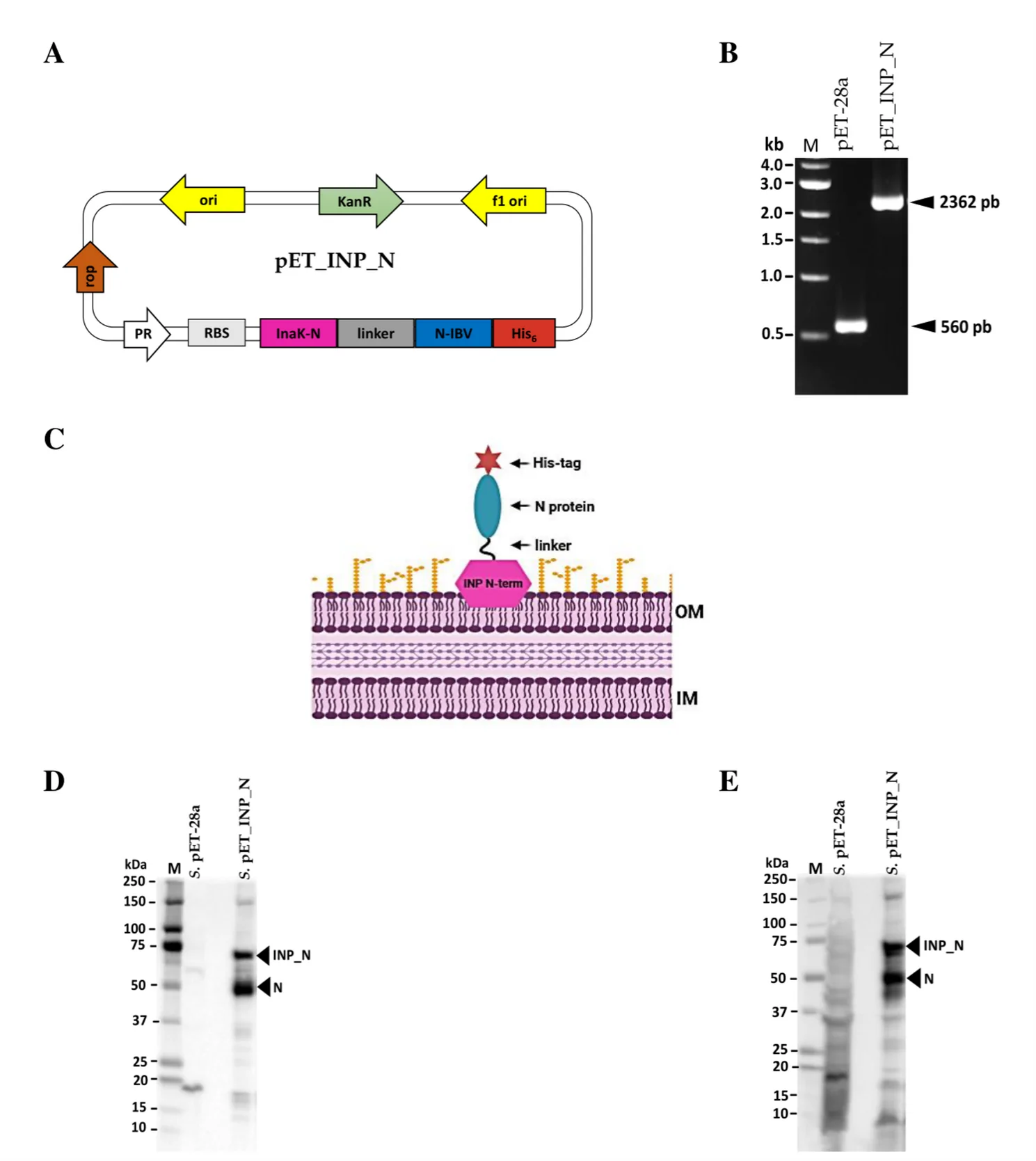
Design and expression of the INP_N fusion construct. **A** Schematic representation of the pET_INP_N plasmid showing the INP_N expression cassette, including the PR promoter, INP N-terminal region, a flexible linker, IBV N protein, and C-terminal 6×His tag. **B** Agarose gel electrophoresis showing a 2,362 bp band corresponding to the expression cassette in pET_INP_N and a 560 bp band corresponding to the empty pET-28a vector. DNA molecular weight marker (kb ladder) is shown in lane M. **C** Schematic representation of the proposed topology of the INP_N fusion protein, based on the reported anchoring properties of the N-terminal domain of INP, with the IBV N protein and C-terminal His-tag expected to be exposed at the bacterial surface. OM, outer membrane; IM, inner membrane. **D** Detection of INP_N (~ 67 kDa) in total protein extracts using an anti-His antibody. **E** Detection of INP_N using a commercial chicken anti-IBV serum. Protein molecular weight markers (kDa) are shown on the left of panels D and E. Equal amounts of total protein were loaded in each lane

An expression cassette encoding the INP_N fusion protein was designed and cloned into the pET-28a vector, generating the recombinant plasmid pET_INP_N. The construct comprised the INP N-terminal domain as a membrane anchor, followed by a flexible linker, the IBV N protein, and a C-terminal 6×His tag (Fig. 1A). A schematic representation of the proposed topology of the INP_N fusion protein is shown in Fig. 1C. Both the pET-28a and pET_INP_N plasmids were transformed into *Salmonella* and verified by PCR amplification of 560 bp and 2362 bp fragments, respectively (Fig. 1B). Western blot analysis confirmed the expression of the ~ 67 kDa fusion protein in *S.* Enteritidis carrying pET_INP_N, detected with both an anti-His antibody (Fig. 1D) and commercial chicken anti-IBV serum (Fig. 1E). Additionally, a band of approximately 50 kDa, corresponding to the predicted molecular weight of the IBV N protein, was observed.

An AlphaFold 3 model of the INP_N fusion protein encoded by the designed construct was generated to obtain a preliminary structural model in the absence of experimentally resolved structures. Structural validation metrics and structural alignment with the crystallized N- and C-terminal domains of the IBV N protein are provided in Additional file 1: Figure S1.

### Effect of INP_N expression on *Salmonella* growth

**Fig. 2:**
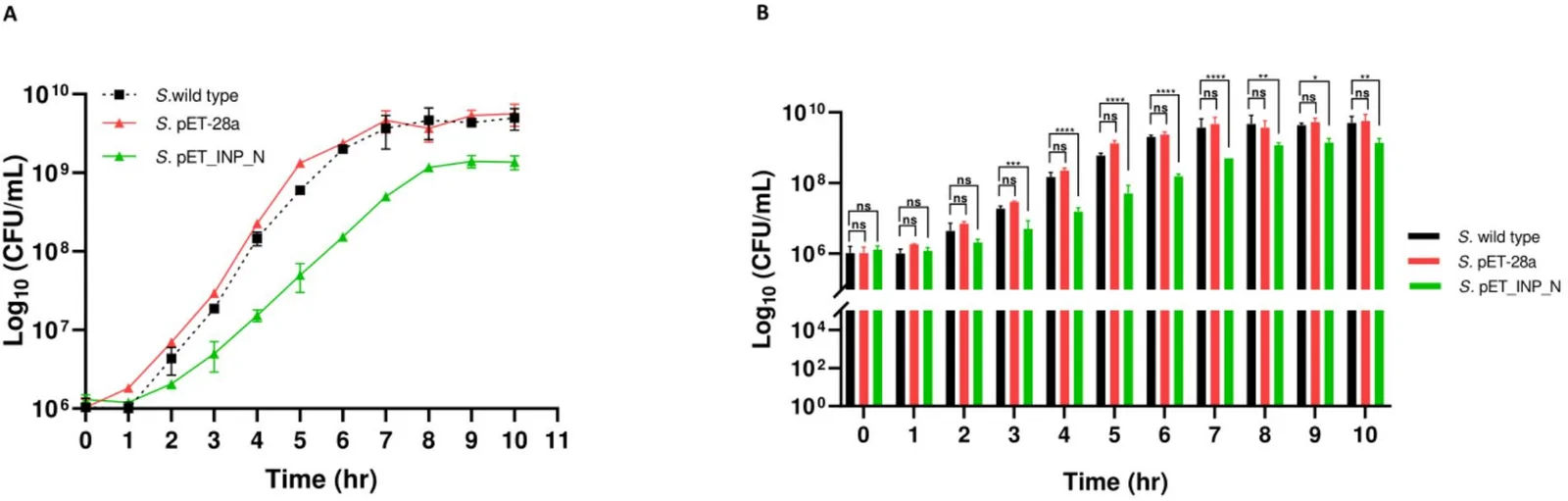
Bacterial growth of WT and recombinant *Salmonella* strains assessed by viable cell counts. **A** CFU/mL measured at hourly intervals over a 10 h cultivation period. Data represent the mean of three biological replicates ± standard deviation (SD). **B** Comparison of CFU/mL among *Salmonella* WT, *S.* pET_INP_N, and *S.* pET-28a strains. A two-way ANOVA with multiple comparisons was performed. Statistical significance levels are indicated as follows: *p < 0.05; **p < 0.01; ***p < 0.001; ****p < 0.0001; ns, not significant (*p* > 0.05)

To evaluate the effect of plasmid carriage and INP_N expression on bacterial growth, CFU/mL were quantified over a 10 h cultivation period for *Salmonella* WT, *S.* pET-28a, and *S.* pET_INP_N (Fig. 2A). No significant differences in CFU/mL were observed between the WT and *S.* pET-28a strains throughout the experiment, with both reaching 5 × 10⁹ CFU/mL at T10. In contrast, *S.* pET_INP_N exhibited significantly lower CFU/mL than the WT strain from T3 onwards (*p* < 0.05), with the greatest differences observed between T3 (5 × 10⁶ vs. 2 × 10⁷ CFU/mL) and T7 (5 × 10⁸ vs. 4 × 10⁹ CFU/mL), as shown in Fig. 2B. Although this difference became less pronounced after T7, *S.* pET_INP_N consistently maintained lower cell densities, reaching 1 × 10⁹ CFU/mL at T10, whereas both WT and *S.* pET-28a reached 5 × 10⁹ CFU/mL.

### Surface exposure of the INP_N protein in *Salmonella*

**Fig. 3:**
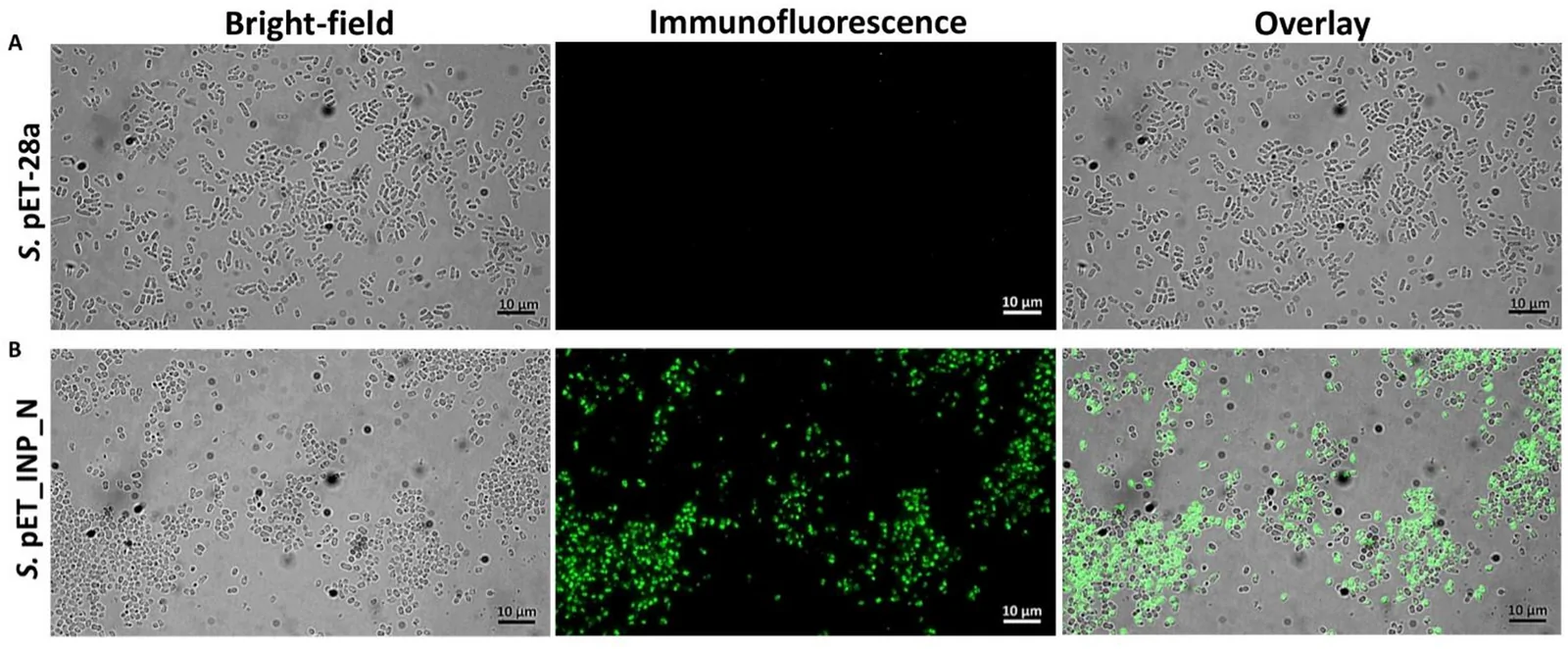
Immunofluorescence detection of His-tagged INP_N in *S.* pET-28a (A) and *S*. pET_INP_N (B). Cells were fixed without permeabilization and incubated with an anti-His monoclonal antibody, followed by an Alexa Fluor 488-conjugated anti-mouse secondary antibody. Panels show bright-field, Immunofluorescence and overlay images. The scale bar represents 10 μm

To investigate the surface exposure of the INP_N fusion protein in the *S.* pET_INP_N strain, immunofluorescence and flow cytometry assays were performed using non-permeabilized cells. Because the fusion protein contains a C-terminal His-tag, anti-His antibodies were used for detection. Immunofluorescence analysis revealed a clear green fluorescence signal associated with *S.* pET_INP_N cells (Fig. 3B), whereas no fluorescence signal was detected in the control strain (*S.* pET-28a) processed under identical conditions (Fig. 3A).

**Fig. 4:**
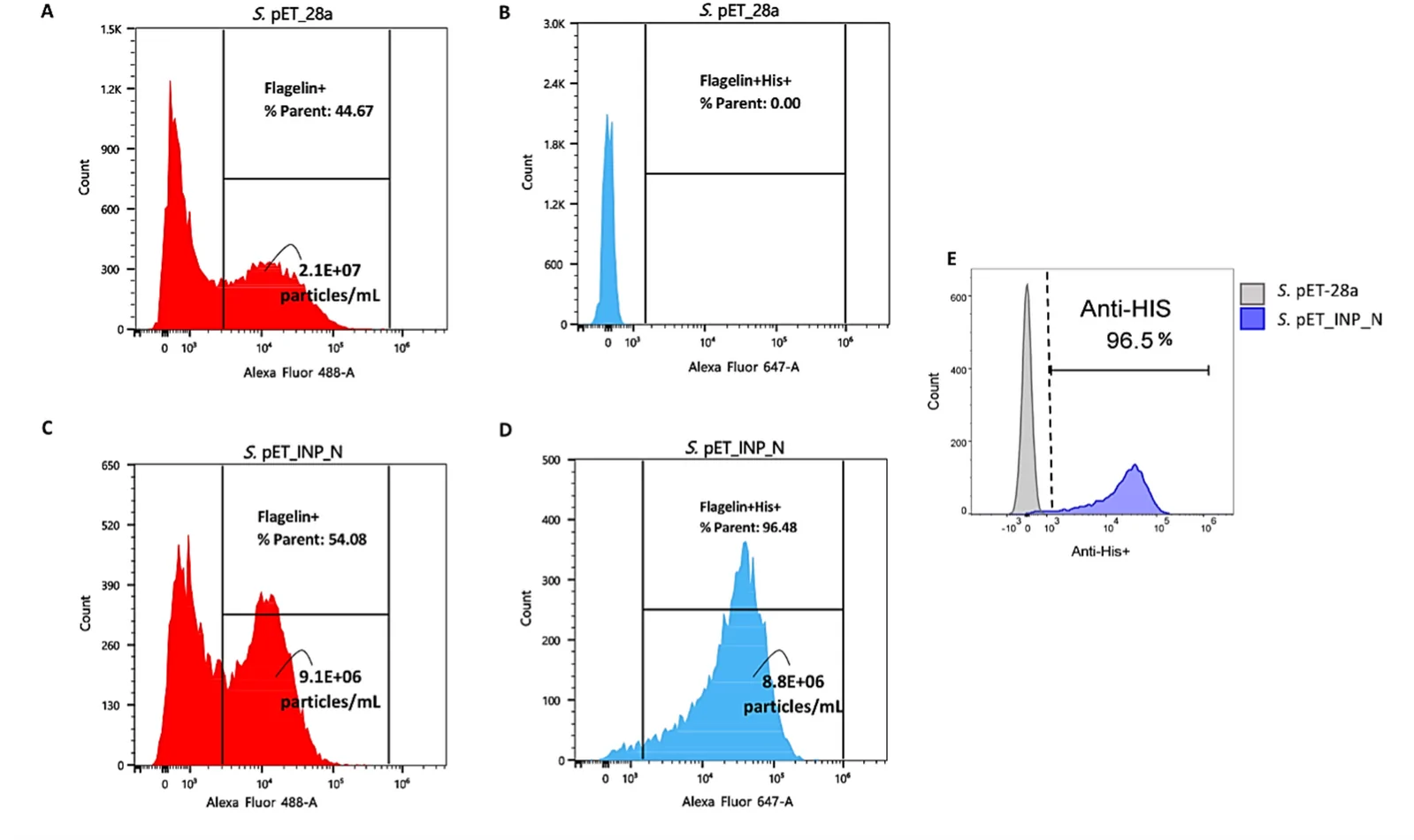
Quantitative flow cytometry of flagellin- and anti-His-positive *S.* pET-28a (control) and *S.* pET_INP_N cells. Histograms showing flagellin-positive populations of *S.* pET-28a (**A**) and *S.* pET_INP_N (**C**). Histograms showing anti-His-positive populations of *S.* pET-28a (**B**) and *S.* pET_INP_N (**D**). Representative overlay histogram comparing anti-His fluorescence intensity distributions of *S.* pET-28a (gray) and *S.* pET_INP_N (blue) cells (**E**). The anti-His-positive gate was defined using the control strain, and 96.5% of *S.* pET_INP_N cells were detected within this gate

**Fig. 5:**
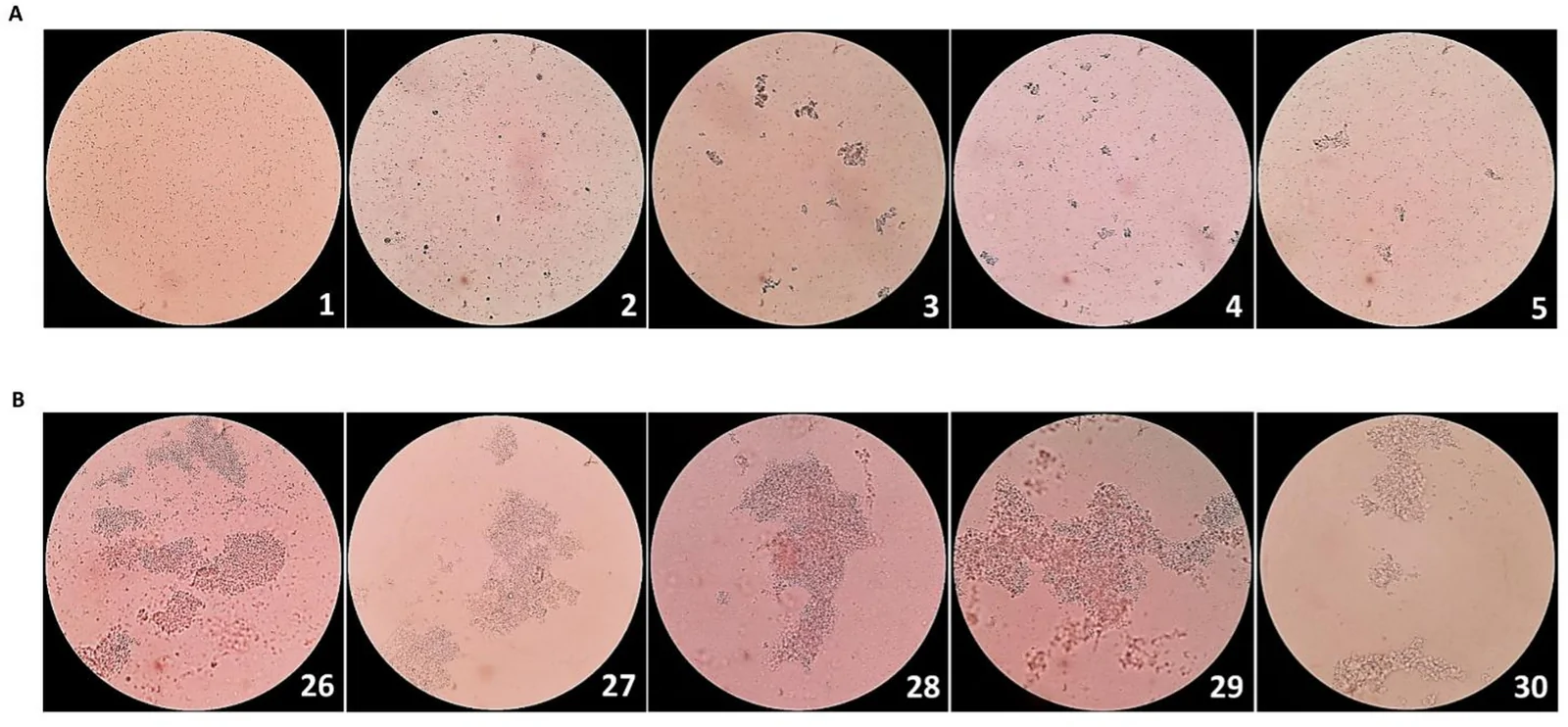
Microscopic images showing microagglutination patterns of *S.* pET_INP_N incubated with IBV-positive and IBV-negative sera. **A** Images of negative microagglutination with IBV-negative sera. **B** Positive microagglutination patterns of recombinant *Salmonella* treated with IBV-positive sera. Total magnification 400×

The proportion of anti-His-positive *Salmonella* cells was determined by flow cytometry. Initial staining with the anti-flagellin antibody defined the bacterial populations of *S.* pET-28a (2.1 × 10⁷ cells/mL) and *S.* pET_INP_N (9.1 × 10⁶ cells/mL) (Fig. 4, panel A, C). Subsequent analysis using the anti-His antibody revealed that 96.5% (8.8 × 10⁶ cells/mL) of the *S.* pET_INP_N population fell within the anti-His-positive gate, whereas no anti-His-positive population was detected in the control strain (Fig. 4, panel B, D). The corresponding fluorescence intensity distributions are shown in the overlay histogram (Fig. 5E).

Flagellin-positive populations of *S.* pET-28a and *S.* pET_INP_N (Additional file 1: Figures S2, panel A, C), as well as the anti-His-positive populations (Additional file 1: Figures S2, panel B, D), were also clearly identified in the representative dot plots used for the gating strategy.

### Diagnostic performance of the slide microagglutination assay using *S.* pET_INP_N as antigen

Inactivated *S.* pET_INP_N cells were evaluated as whole-cell antigens in a slide microagglutination assay for the detection of anti-IBV antibodies. All 50 hen sera characterized by ELISA (25 positive and 25 negative) were tested by microagglutination. Within this proof-of-concept study, all ELISA-positive sera were also positive by microagglutination, showing agreement for this subset. In contrast, among the 25 ELISA-negative sera, 20 exhibited negative agglutination patterns, whereas 5 showed positive reactions (Additional file 1: Table S1, Figure S3). Representative images at 400× magnification illustrate the absence (Fig. 5A) or presence (Fig. 5B) of microagglutination.

The results from the 50 sera tested by both methods were summarized in a 2 × 2 contingency table (Table 1). The microagglutination assay showed a sensitivity of 100% (95% CI 87–100%) and a specificity of 80% (95% CI 61–91%). The PPV was 83.3% (95% CI 66–93%), and the NPV was 100% (95% CI 84–100%). These diagnostic performance measures are summarized in Table 2. Concordance between the two assays yielded a Cohen’s kappa index of 0.80, consistent with substantial agreement (Table 3).

**Table 1:** Results of the analysis of IBV positive and negative sera, by Elisa and microagglutination test

Diagnostic techniques		ELISA		Total
		Positive (+)	Negative (-)	
Mat	Positive (+)	25	5	30
	Negative (-)	0	20	20
Total		25	25	50

**Table 2:** Diagnostic performance measures of the slide microagglutination test with 95% confidence intervals

	Mat	95% CI
Sensitivity (%)	100	86.7–100
Specificity (%)	80	60.9–91.1
Positive predictive value (PPV)	83.3	66.4–92.7
Negative predictive value (NPV)	100	83.9–99.9

**Table 3:** Concordance between slide microagglutination assay and ELISA

		Value	Approx. Sig
Measure of agreement	Kappa coefficient	0.80	0
N of valid cases		50	

**Fig. 6:**
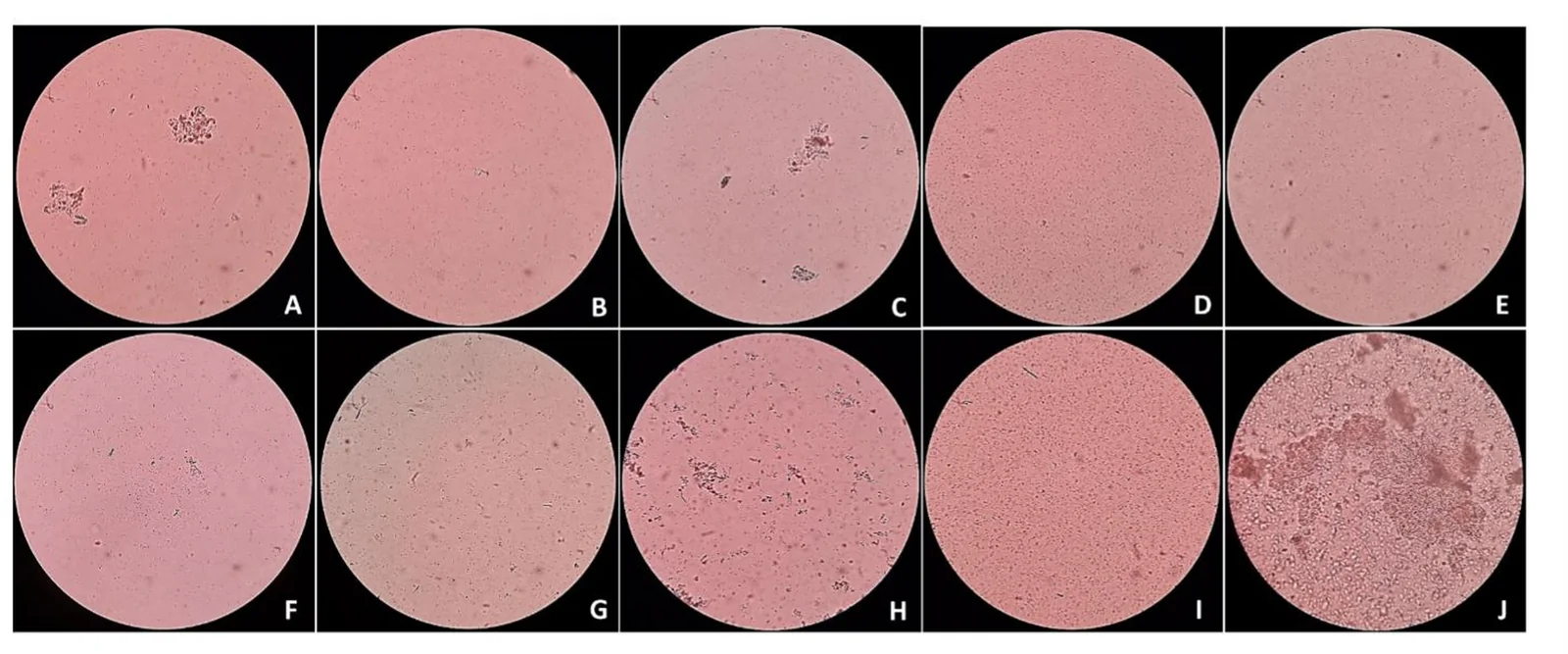
Bright-field microscopy images showing microagglutination patterns of *S.* pET_INP_N incubated with ten commercial chicken antisera. **A–I** No agglutination observed with antisera against Marek’s Disease Virus (**A**), Fowl Adenovirus Type I (**B**), Newcastle Disease Virus (**C**), *M. synoviae* (**D**), Infectious Laryngotracheitis Virus (**E**), Infectious Bursal Disease Virus (**F**), Avian Influenza A (H5N2) Virus (**G**), Fowlpox Virus (**H**), and Chicken Anemia Virus (**I**). **J** Positive microagglutination observed with antiserum against IBV (Massachusetts strain). Images captured at 400× total magnification

Cross-reactivity of the surface display platform with avian pathogens associated with infectious bronchitis and other major avian diseases was evaluated using nine commercial chicken antisera specific for Fowl Adenovirus Type I (FAdV-I), Chicken Anemia Virus (CAV), Fowlpox Virus (FPV), *M. synoviae*, Avian Influenza A (H5N2) Virus (AIV H5N2), Infectious Laryngotracheitis Virus (ILTV), Newcastle Disease Virus (NDV), Marek’s Disease Virus (MDV), and Infectious Bursal Disease Virus (IBDV). None of these antisera induced microagglutination of *S.* pET_INP_N. In contrast, the commercial IBV-positive antiserum produced a strong microagglutination reaction (Table 4; Fig. 6).

Additionally, all sera that tested positive in the slide microagglutination assay (25 true positives and 5 false positives) were negative for anti-*Salmonella* antibodies by commercial ELISA (Additional file 1: Table S2).

**Table 4:** Slide microagglutination assay results obtained with *S.* pET_INP_N strain using different serum

Serum type	Number of samples (*n*)	S. pET_INP_N microagglutination
IBV-positive sera	25	25/25 (100%)
IBV-negative sera	25	5/25 (20%)
Commercial chicken anti-IBV serum	1	1/1 (100%)
Commercial antisera to other bacterial and viral pathogens (NDV, ILTV, MDV, CAV, IBDV, AIV H5N2, FPV, FAdV-I and *M. synoviae*)	9	0/9 (0%)

## Discussion

Microagglutination is a sensitive, rapid, and cost-effective serological assay widely used for disease diagnosis and surveillance in animals and humans [3]. However, its conventional use is primarily limited to bacterial pathogen detection and requires live cultures, posing biosafety risks. Bacterial surface display systems offer a promising alternative, as they enable the presentation of heterologous proteins or peptides on the bacterial surface and have diverse diagnostic applications [12, 14, 41]. However, integration of this technology into slide-based microagglutination assays has not yet been explored. In this study, we present a proof-of-concept for using an attenuated *S.* Enteritidis surface-display platform expressing the IBV N protein as a model antigen for antibody detection by slide microagglutination. The assay showed preliminary reactivity and substantial agreement with the reference ELISA.

The presence of the empty pET-28a plasmid did not significantly affect CFU/mL in *S.* pET-28a compared with the WT strain, despite being a high-copy-number vector, suggesting that plasmid carriage did not impose a detectable metabolic burden under the conditions tested. This observation contrasts with previous reports describing growth impairment associated with the maintenance of high-copy-number plasmids [42]. In contrast, expression of the INP_N resulted in reduced CFU/mL in the recombinant strain (*S.* pET_INP_N) compared with the WT strain, particularly between T3 and T7. This effect is consistent with the additional metabolic burden associated with heterologous protein expression, including increased demands for energy and biosynthetic precursors during transcription and translation [17, 43–46]. This reduction in CFU/mL could have implications for large-scale cultivation processes, where a balance between biomass accumulation and recombinant protein expression is required to maximize antigen yield, as previously reported for recombinant expression systems [47–49]. Future studies could explore optimized cultivation strategies to improve bacterial performance during heterologous expression.

Western blot revealed a ~ 67 kDa band corresponding to the INP_N fusion protein and an additional ~ 50 kDa band compatible with the N protein lacking the anchoring motif. This may reflect partial cleavage of the chimera, potentially related to the susceptibility of the anchor domain to proteolytic cleavage [18, 43] or degradation under cellular stress conditions associated with high-level recombinant protein expression [50, 51], elevated temperatures, or partially denaturing environments [52]. Despite this, the intact fusion protein was prominently detected; however, further optimization of the culture conditions for recombinant protein expression is still required.

Immunofluorescence and flow cytometry analyses performed on fixed, non-permeabilized cells showed specific anti-His labeling in *S.* pET_INP_N, whereas no signal was detected in the control strain. Flow cytometry further indicated that 96.5% of the *S.* pET_INP_N cell population was anti-His positive. These results indicate that the C-terminal His-tag of the fusion protein is accessible to antibody detection in non-permeabilized cells, supporting the surface exposure of the recombinant protein in the *S.* pET_INP_N strain. This observation is consistent with previous studies describing the use of the N-terminal domain of INP as an anchoring motif for heterologous proteins in Gram-negative bacteria [17, 19, 43, 53, 54].

The slide microagglutination assay using inactivated *S.* pET_INP_N cells showed a preliminary sensitivity of 100% (95% CI: 87–100%), as all ELISA-IBV-positive sera were also positive in the assay, with no false-negative results observed. While specificity was 80% (95% CI: 61–91%), with five false-positive sera detected. These results indicate that the assay correctly identified all positive samples within this proof-of-concept study, while the specificity estimate indicated that the assay correctly classified most negative sera. However, the relatively wide confidence interval suggests imprecision in the estimate, likely due to the limited sample size [55, 56]. Evaluation using a larger serum panel would allow a more precise estimate of the assay’s performance characteristics.

The observed reduction in specificity may be associated with non-specific interactions between serum components and the bacterial platform, contributing to false-positive reactions. In whole-cell bacterial assays, such interactions may be mediated by naturally occurring antibodies or proteins with polyreactive binding capacity [57–59], which could interact with bacterial surface structures or with determinants unrelated to the recombinant antigen, contributing to background microagglutination. In this context, inclusion of a control strain lacking the recombinant antigen would help to distinguish responses associated with the bacterial platform from those specifically directed against the recombinant antigen, thereby providing further insight into the antigen-specific contribution to the microagglutination response. In addition, optimization strategies such as surface blocking or serum pretreatment could further reduce background reactivity and improve assay performance.

Despite the observed reduction in specificity, the microagglutination assay showed substantial agreement with the reference ELISA (κ = 0.80) [60], indicating consistent classification between both methods under the evaluated conditions.

Notably, the *S.* pET_INP_N strain did not produce positive microagglutination reactions when tested against antisera directed against FAdV-I, CAV, FPV, *M. synoviae*, AIV (H5N2), ILTV, NDV, MDV, and IBDV. These pathogens are frequently reported in poultry production systems and are commonly included in differential diagnostic investigations of infectious diseases in chickens [61–66]. These results suggest that, under the conditions evaluated, no cross-reactivity was detected with the heterologous antisera panel. However, evaluation using field sera from chickens naturally exposed to various common avian pathogens, such as NDV, *Mycoplasma*, H5N1, and *Salmonella*, will be necessary to assess assay performance under field conditions and to further clarify its effectiveness, limitations, and the potential impact of interference or cross-reactivity on assay specificity.

Unlike ELISA and colloidal gold immunochromatographic assays, which often require recombinant antigen purification before immobilization onto microplates or conjugation to gold nanoparticles, respectively [67–70], the proposed approach displays the recombinant antigen on the bacterial surface, thereby avoiding the need for the purification step and simplifying downstream processing during reagent preparation. Although the impact of this simplified workflow on production costs and scalability was not assessed in the present study, these aspects warrant further investigation during future process optimization.

The preliminary performance of the assay was generally within the range reported for IBV serological assays. The observed sensitivity was within the range reported for ELISA (70–100%) and was numerically higher than values reported in immunochromatographic studies (91–92%), whereas specificity (80%) was within the range reported for ELISA (77–100%) and near the lower values reported for immunochromatographic assays (81–97%) [71–75]. Because these comparisons are based on independent studies with different serum panels and experimental designs, they should be interpreted cautiously.

In addition, the assay allowed direct visual interpretation within approximately 5–8 min, comparable to the turnaround time reported for immunochromatographic assays (10 min) and shorter than that of conventional ELISA (1–2 h), which typically requires multiple incubation and washing steps together with specialized equipment for signal detection [74, 76–78].

Previous studies have demonstrated the use of the N-terminal domain of INP for the surface display of different heterologous proteins in Gram-negative bacteria [17, 18, 43, 53, 79]. Based on these reports, the platform could be adapted to other viral, bacterial, or parasitic antigens by replacing the heterologous coding sequence while maintaining the same anchoring strategy. However, such applicability remains to be experimentally validated, as antigen size, folding, epitope accessibility, and expression levels can influence surface display efficiency [14, 15].

An additional consideration is biosafety. In this proof-of-concept study, an attenuated *Salmonella* strain engineered to display a selected viral antigen was used. Prior to microagglutination assays, the recombinant cells were inactivated using paraformaldehyde, a chemical commonly used in diagnostic reagent preparation due to its ability to preserve surface antigen integrity while eliminating bacterial viability [80–82]. This inactivation approach is relevant for bacterial surface-display systems, where biosafety, containment, and environmental release are important considerations [83–86]. However, evaluation of biological containment, genetic stability, and regulatory compliance was beyond the scope of this study [87–89], and will be required for future implementation of the platform.

Future work may focus on the exploration of blocking strategies to reduce non-specific interactions and improve assay specificity, as well as further optimization of the generated platform to enhance antigen display efficiency. In addition, evaluation in larger and more diverse cohorts, including field-derived samples, will be important to further assess the performance and translational potential of the system. The development of freeze-dried formulations could enhance reagent stability and facilitate storage and transport under non-ideal conditions.

## Conclusions

Our findings support the preliminary feasibility of using an attenuated *Salmonella* surface-display platform in a slide microagglutination assay for antibody detection. This whole-cell approach reduces the need for pathogen cultivation, antigen purification, and chemical conjugation steps. However, further optimization and evaluation under field conditions are required to further establish its diagnostic performance.

## Supplementary Information

Below is the link to the electronic supplementary material.

Supplementary Material 1.

## Data Availability

The datasets supporting the conclusions of this article are included within the article and its supplementary information file.

## Abbreviations

IBV: Infectious bronchitis virus
INP: Ice nucleation protein
N: Nucleocapsid
ELISA: Enzyme-linked immunosorbent assay
DNA: Deoxyribonucleic acid
CRD: Central repeat domain
PCR: Polymerase chain reaction
LB: Luria-Bertani
His-tag: Histidine tag
JCAT: Java codon adaptation tool
pTM: Predicted template modeling score
ipTM: Interface predicted template modeling score
RMSD: Root mean square deviation
PDB: Protein data bank
WT: Wild type
DTT: Dithiothreitol
RT: Room temperature
PMSF: Phenylmethylsulfonyl fluoride
EDTA: Ethylenediaminetetraacetic acid
PAGE: Polyacrylamide gel electrophoresis
DPBS: Dulbecco’s phosphate-buffered saline
PFA: Paraformaldehyde
FACS: Fluorescence activated cell sorting
HRP: Horseradish peroxidase
BSA: Bovine serum albumin
SPF: Specific pathogen-free
LPS: Lipopolysaccharide
PPV: Positive predictive value
NPV: Negative predictive value
CI: Confidence intervals
OD: Optical density
CFU: Colony-forming units per millilitre
TBST: Tris-buffered saline with Tween
INP_N: fusion protein formed by ice nucleation protein domain and nucleocapsid protein
pET_INP_N: pET-28a plasmid containing the fusion protein expression cassette
*S.* pET-28a: *Salmonella* Enteritidis carrying the empty pET-28a plasmid
*S.* pET_INP_N: *Salmonella* Enteritidis carrying the pET_INP_N plasmid
