# Supplementary material for: Whole-cell surface-display system in attenuated Salmonella Enteritidis for slide microagglutination-based antibody detection
Source: Microb Cell Fact. 2026 Aug 8;25:210. doi: 10.1186/s12934-026-03083-x (PMC13660097; doi:10.1186/s12934-026-03083-x)
Supplement: Supplementary file 1 [file 12934_2026_3083_MOESM1_ESM.docx]

Additional file 1

## Structural similarity between the INP_N N protein domain and IBV N protein structures

The 544-aa INP_N construct was modeled using AlphaFold 3. Five structural models were generated, and the top-ranked model was selected (Figure S1, panel C). This model exhibited a pTM score of 0.35 and a QMEANDisCo global score of 0.48. Low-confidence regions were identified at residues 71–95, 111–229, 358–416, and 532–614. Structural validation yielded a ProSA-web Z-score of –6.61 (Figure S1, panel A) and a Ramachandran plot showing 74% of residues in favored regions and 26% in additionally allowed regions (Figure S1, panel B).

Despite these limitations, structural alignment showed correspondence between the IBV N protein domain extracted from the INP_N fusion model and crystallized N- and C-terminal domains of the IBV N protein, with TM-scores of 0.91 and 0.98, and RMSD values of 1.39 Å and 0.57 Å, respectively (Figure S1, panel D).

**Figure S1.** Quality assessment of the predicted INP_N model and structural alignment of the N protein. **(A)** Overall quality plot showing that the predicted model (indicated by a black dot) falls within the range of experimentally determined structures (blue shaded area). **(B)** Ramachandran plot indicating that 74% of the residues are located in the most favored regions [A, B, L] (red area), while the remaining 26% are in additionally allowed regions [a, b, l, p] (brown area). **(C)** Predicted model of INP_N, where the extracellular region of INP N-terminal domain is highlighted in pink, the flexible linker is indicated in blue, the full-length N protein is shown in light blue, and the His-tag is depicted in red. **(D)** Structural alignment of the N protein model with the crystallized N-terminal (yellow) and C-terminal (green) domains of the IBV N protein.

**Figure S2**. Gating strategy used to evaluate surface exposure of the fusion protein INP_N in S. pET_INP_N cells. (**A)** S. pET-28a population positive for flagellin. (**B)** S. pET-28a population positive for His-tag. **(C)** S. pET_INP_N population positive for flagellin. **(D)** S. pET_INP_N population positive for His-tag.

**Figure S3**. Bright-field microscopy images showing microagglutination patterns of S. pET_INP_N incubated with IBV-positive and IBV-negative sera. (**A)** *S.* pET_INP_N treated with IBV-negative sera. (**B)** *S.* pET_INP_N treated with IBV-positive sera. Total magnification 400x.

**Table S1.** Results of the anti-IBV antibody detection test by ELISA and microagglutination test.

| N°. of serum tested | Result | |
| --- | --- | --- |
|  | **ELISA** | **Microagglutination** |
| 1 | - | - |
| 2 | - | - |
| 3 | - | - |
| 4 | - | - |
| 5 | - | - |
| 6 | - | **+** |
| 7 | - | **+** |
| 8 | - | - |
| 9 | - | - |
| 10 | - | - |
| 11 | - | - |
| 12 | - | **+** |
| 13 | - | - |
| 14 | - | **+** |
| 15 | - | - |
| 16 | - | - |
| 17 | - | - |
| 18 | - | - |
| 19 | - | - |
| 20 | - | - |
| 21 | - | - |
| 22 | - | **+** |
| 23 | - | - |
| 24 | - | - |
| 25 | - | - |
| 26 | **+** | **+** |
| 27 | **+** | **+** |
| 28 | **+** | **+** |
| 29 | **+** | **+** |
| 30 | **+** | **+** |
| 31 | **+** | **+** |
| 32 | **+** | **+** |
| 33 | **+** | **+** |
| 34 | **+** | **+** |
| 35 | **+** | **+** |
| 36 | **+** | **+** |
| 37 | **+** | **+** |
| 38 | **+** | **+** |
| 39 | **+** | **+** |
| 40 | **+** | **+** |
| 41 | **+** | **+** |
| 42 | **+** | **+** |
| 43 | **+** | **+** |
| 44 | **+** | **+** |
| 45 | **+** | **+** |
| 46 | **+** | **+** |
| 47 | **+** | **+** |
| 48 | **+** | **+** |
| 49 | **+** | **+** |
| 50 | **+** | **+** |

**Table S2.** Optical density values and S/P ratios obtained with a commercial anti-Salmonella ELISA in serum samples positive by microagglutination.

| Sample ID | D.O. | S/P ratio | Result |
| --- | --- | --- | --- |
| Negative control | 0.085 |  | Negative |
| Negative control | 0.085 |  | Negative |
| Positive control | **0.808**^a^ |  | **Positive** |
| Positive control | **0.790**^a^ |  | **Positive** |
| Positive serum 1 | 0.087 | 0.003 | Negative |
| Positive serum 2 | 0.088 | 0.004 | Negative |
| Positive serum 3 | 0.089 | 0.006 | Negative |
| Positive serum 4 | 0.092 | 0.010 | Negative |
| Positive serum 5 | 0.092 | 0.010 | Negative |
| Positive serum 6 | 0.090 | 0.007 | Negative |
| Positive serum 7 | 0.088 | 0.004 | Negative |
| Positive serum 8 | 0.096 | 0.015 | Negative |
| Positive serum 9 | 0.089 | 0.006 | Negative |
| Positive serum 10 | 0.096 | 0.015 | Negative |
| Positive serum 11 | 0.091 | 0.008 | Negative |
| Positive serum 12 | 0.090 | 0.007 | Negative |
| Positive serum 13 | 0.098 | 0.018 | Negative |
| Positive serum 14 | 0.090 | 0.007 | Negative |
| Positive serum 15 | 0.092 | 0.010 | Negative |
| Positive serum 16 | 0.090 | 0.007 | Negative |
| Positive serum 17 | 0.091 | 0.008 | Negative |
| Positive serum 18 | 0.092 | 0.010 | Negative |
| Positive serum 19 | 0.091 | 0.008 | Negative |
| Positive serum 20 | 0.088 | 0.004 | Negative |
| Positive serum 21 | 0.087 | 0.003 | Negative |
| Positive serum 22 | 0.090 | 0.007 | Negative |
| Positive serum 23 | 0.092 | 0.010 | Negative |
| Positive serum 24 | 0.091 | 0.008 | Negative |
| Positive serum 25 | 0.087 | 0.003 | Negative |
| Negative serum 5 | 0.091 | 0.008 | Negative |
| Negative serum 9 | 0.092 | 0.010 | Negative |
| Negative serum 10 | 0.093 | 0.011 | Negative |
| Negative serum 17 | 0.094 | 0.013 | Negative |
| Negative serum 19 | 0.086 | 0.001 | Negative |

^a^ Positive Cutoff (S/P) ≥ 0.5
